# Evidence for the Prevention and Treatment of Stroke in Dialysis Patients

**DOI:** 10.1111/sdi.12281

**Published:** 2014-07-07

**Authors:** William Herrington, Richard Haynes, Natalie Staplin, Jonathan Emberson, Colin Baigent, Martin Landray

**Affiliations:** * Clinical Trial Service Unit and Epidemiological Studies Unit (CTSU), Nuffield Department of Population Health, University of OxfordOxford, United Kingdom; † Oxford Kidney Unit, Churchill Hospital, Oxford University Hospitals NHS TrustOxford, United Kingdom

## Abstract

The risks of both ischemic and hemorrhagic stroke are particularly high in dialysis patients of any age and outcomes are poor. It is therefore important to identify strategies that safely minimize stroke risk in this population. Observational studies have been unable to clarify the relative importance of traditional stroke risk factors such as blood pressure and cholesterol in those on dialysis, and are affected by biases that usually make them an inappropriate source of data on which to base therapeutic decisions. Well-conducted randomized trials are not susceptible to such biases and can reliably investigate the causal nature of the association between a potential risk factor and the outcome of interest. However, dialysis patients have been under-represented in the cardiovascular trials which have proven net benefit of commonly used preventative treatments (e.g., antihypertensive treatments, low-dose aspirin, carotid revascularization, and thromboprophylaxis for atrial fibrillation), and there remains uncertainty about safety and efficacy of many of these treatments in this high-risk population. Moreover, the efficacy of renal-specific therapies that might reduce cardiovascular risk, such as modulators of mineral and bone disorder, online hemodiafiltration, and daily (nocturnal) hemodialysis, have not been tested in adequately powered trials. Recent trials have also demonstrated how widespread current practices could be causing stroke. Therefore, it is important that reliable information on the prevention and treatment of stroke (and other cardiovascular disease) in dialysis patients is generated by performing large-scale randomized trials of many current and future treatments.

## Stroke Definition and Subtypes

Stroke is an important cause of disability and the third leading cause of death worldwide (1–4). Stroke can be defined clinically by the rapid development of a focal or global disturbance of cerebral function, lasting for at least 24 hours (unless interrupted by death), with no apparent nonvascular cause. Cerebrovascular disease is a very heterogeneous disease and is classified by the different pathologies. The three principal stroke subtypes are cerebral infarction (or ischemic stroke), primary intracerebral hemorrhage, and subarachnoid hemorrhage. In the United States (US) general population, these subtypes make up 87%, 10%, and 3% of strokes, respectively (5).

Ischemic stroke can be further subdivided by etiology using the modified Trial of Org 10172 in Acute Stroke Treatment (TOAST) criteria into large vessel, cardioembolic, and small vessel (or lacunar) strokes (6). Cerebral infarction may also result from other insults which are not always considered in ischemic stroke subtype classifications, such as severe hemodynamic or metabolic disturbances. Primary intracerebral hemorrhage can be subclassified anatomically into deep or lobar subtypes (7). The majority of subarachnoid hemorrhages (which are not a focus of this review) are caused by cerebral aneurysm rupture (8).

The heterogeneity of stroke etiology means that the risk factors and treatments for each subtype might be expected to differ. Consequently, to study individual ischemic and hemorrhagic stroke subtypes requires both detailed stroke characterization and a very large number of events. Most studies are restricted to considering only the main stroke subtypes.

## Stroke Risk and Outcomes in Those on Dialysis

**Fig. 1:**
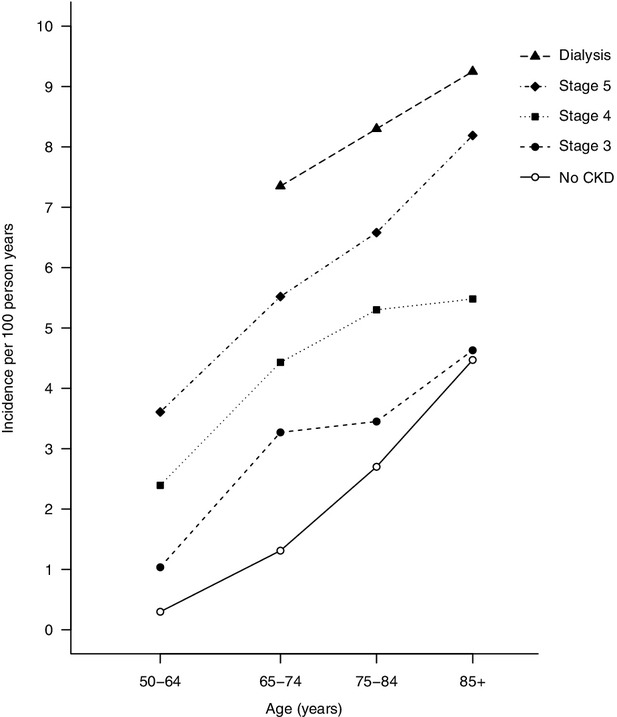
Rates of incident stroke per 100 person years in prevalent chronic kidney disease patients in the United States in 2006 by age. Adapted from the 2009 United States Renal Data System annual report (10); CKD, chronic kidney disease; stage 5 includes all those with an eGFR <15 ml/minute/1.73 m^2^ not on dialysis; data in dialysis patients aged <65 years unavailable. Note that the interpretation and reporting of these data are the responsibility of the authors and in no way should be seen as an official policy or interpretation of the US government.

Chronic kidney disease (CKD) is associated with an increased risk of vascular events, including stroke (9). There is a clear relationship between worsening renal function and stroke incidence, with patients on dialysis at the highest risk (Fig. 1). In the United States, the risk of stroke among those on dialysis is between two- and seven times higher than in those without kidney disease (10). This reflects a similar increase in the risk of ischemic and hemorrhagic stroke (11). Across all age groups, those on dialysis have consistently greater absolute risk of a stroke than people of a similar age not on dialysis. The relative increased risk of stroke is particularly high among young dialysis patients. In a large study from Taiwan, incidence of both ischemic and hemorrhagic stroke in those on dialysis aged less than 45 was at least 10 times greater than the general population. The very high risk in young dialysis patients means the relationship between age and stroke in dialysis patients is less steep than the relationship observed in the general population (12).

Outcomes after stroke in dialysis patients are also worse than in general populations. In the United States, end-stage renal disease is associated with a 3-fold increase in in-hospital mortality following a stroke (13), while dialysis patients in Taiwan with stroke have a four- to five times higher mortality rate than age- and sex-matched stroke patients not on dialysis (12). In the United States, around two-thirds of those on dialysis over 75 years of age will be dead within a year of a stroke (compared to about a quarter of those without a stroke) (10), and stroke is responsible for one in 15 dialysis deaths (10). It is therefore important to identify treatments that safely reduce stroke risk among dialysis patients of all ages.

In this review, we discuss the role of blood pressure, the current evidence for the prevention of atherosclerotic and thromboembolic stroke, the efficacy of antiplatelet therapy, the safety of thrombolysis, and the potential for renal-specific treatments to modify stroke risk in dialysis patients, with an emphasis on the evidence generated from large-scale randomized trials.

## Stroke Risk Factors

Diabetes mellitus and hypertension are important causes of both end-stage renal disease and stroke (14,15). The increased stroke risk in dialysis patients may therefore simply reflect the increased prevalence of “traditional” stroke risk factors. However, it has also been proposed that “emerging” stroke risk factors such as markers of CKD–mineral and bone disorder (16,17), inflammatory biomarkers, (18) and uremic toxins, which are all disturbed in CKD, may contribute. The use of erythropoiesis-stimulating agents (ESAs) also increases the risk of stroke (19).

Table 1 summarizes some of the available observational studies of stroke in dialysis populations. In these studies, older age and diabetes are consistently observed to be independent stroke risk factors (12,18,20–22). However, established stroke risk factors, such as high blood pressure, appear to be of less relevance in dialysis patients than would be predicted from observations in general populations. This has led to uncertainty about the precise importance of some traditional stroke risk factors in those on dialysis. However, major biases intrinsic to the study of diseased populations may explain the differences between dialysis and general population studies. Using the examples of blood pressure and cholesterol, the potential for such biases to distort observed associations in dialysis patients is discussed below.

**Table 1:** Published observational data on stroke in selected and unselected dialysis populations

Author (reference) (acronym)	Year	Study design	Location (population)	Ethnicity	Stroke number	Population size/type	Mean Age (years)	Crude incidence (% p.a)	% Ischemic	Case fatality	Independent stroke predictors
Kawamura (108)	1971–1994	R	Miyazaki, Japan	Japanese 100%	80^a^	4064 HD	54	1.2	30	I: 50% H: 71%	–
Kuo (109)	1999–2008	R	Taiwan	Chinese 100%	119	644 HD	54	4.2	80	–	–
Seliger (11)	1993–1998	R	US	Mainly African Americans and Caucasians	32,151^a^	436000 HD+PD	72	3.2–5.9	–	–	–
Seliger (20)	1993–1999	R	US	White 53% Black 40% Asian/other 7%	915^a^	8920 HD+PD	60	3.3^g^	84	–	Older age; high mean predialysis blood pressure; lower serum albumin; considered undernourished
Toyoda (93)	1980–2002	R	Fukuoka, Japan	Japanese 100%	144	1740 HD	62	1.2	60	I: 6% H: 50%	–
Shah (76)	1998–2007	R	Canada (with AF)	–	107	1626 HD+PD	75	3.1	–	–	–
Chan (72)	2003–2007	R	US (with AF)	White 80%^f^ Black 14% Other 6%	102^a^	1671 HD	73	4.8^c^	82	–	Warfarin prescription; higher CHADS2 score
Olesen (69)	1997–2008	R	Denmark (with AF)	–	164^e^	901 RRT	67	5.6	–	–	Older age; prior stroke; no warfarin prescription
Power (22)	2002–2009	R	London, England	White 42% Black 18% S.Asian 34% Other 5%	121	2474 HD	58	1.7	71	I: 7% H: 32%	Diabetes; prior cerebrovascular disease
Wang (12)	1998–2009	R	Taiwan	Chinese 100%	2424	79,986 HD+PD	52	1.7	58	–	Older age; diabetes; hypertension; anemia
Iseki (28,110)	1988–1998	P	Okinawa, Japan	Japanese 100%	259^a^	3741 HD+PD	53	1.7	37	–	Hypertension
Winkelmayer (73)	1994–2006	P	US (≥65 years+AF)^d^	White 67%^d^	188	1185 HD	–	9.5^d^	84^a^^,^^d^	–	–
Wizemann (62)(DOPPS)	1994–2004	P	Worldwide (with AF)	–	148	3250 HD	–	3.4	–	–	Atrial fibrillation
Sozio (21) (CHOICE)	1995–2004	P	US	White 67% Black 28% Other 5%	176	1041 HD+PD	58	4.3	87	I: 28% H: 90%	Older age; white race; diabetes; coexistent diseases
Tripepi (103) (CREED)	1997–2007	P	Southern Italy	–	47	283 HD+PD	61	3.9^f^	85^f^	I: 52% H: 33%^f^	Smoking; higher pulse pressure; higher hemoglobin; older age; higher triglycerides; higher left ventricular mass
Sanchez-Perales (27)	1999–2008	P	Jaen, Spain	–	34	449 HD+PD	64	2.4^b^	–	I: 35%	Older age; atrial fibrillation; diabetes
Shoji (18)	2003–2004	P	Japan	Japanese 100%^f^	1592	45,390 HD	62	3.5	70	I: 8% H: 27%	Lower albumin; male sex; diabetes; higher non-HDL-C; higher CRP; low BMI; older age
Delmez (26) (HEMO)	1995–2000	RCT	US	White/Other 37% Black 63%	63 stroke deaths	1846 HD	58	1.2	73	–	Low albumin; diabetes; higher hemocrit; low BMI
Wanner (111,112) (4D)	1998–2002	RCT	Germany (type 2 diabetes)	Predominately White	103	1225 HD	66	2.1^f^	87	–	Nonsinus rhythm
Fellström (113,114) (AURORA)	2003–2009	RCT	Worldwide	White 85% Black 4% Asian 5% Other 6%	164	2776 HD	64	1.8^f^	71	I: 32% H: 70%	–

p.a., per annum; R, retrospective; P, prospective; RCT, randomized control trial; AF, atrial fibrillation; HD, hemodialysis; PD, peritoneal dialysis; RRT, renal replacement therapy; US, United States; I, ischemic; H, hemorrhagic; BMI, body mass index; HDL-C, high-density lipoprotein cholesterol; CRP, C-reactive protein; USRDS, United States Renal Data System; DOPPS, Dialysis Outcomes and Practice Patterns Study; CHOICE, Choices For Healthy Outcomes in Caring for ESRD; CREED, Cardiovascular Risk Extended Evaluation in Dialysis patients; HEMO, Hemodialysis study; 4D, Die Deutsche Diabetes Dialyse Studie; AURORA, A study to evaluate the Use of Rosuvastatin in subjects On Regular hemodialysis: an Assessment of survival and cardiovascular events.

a May include subarachnoid hemorrhage.

b Ischemic stroke only.

c Includes transient ischemic attacks.

d Data limited to propensity matched cohort.

e Includes stroke and other systemic thromboembolism.

f Estimated.

g Age-standardized incidence.

### Blood Pressure and Stroke

In apparently healthy adults, there is a log-linear relationship between stroke mortality and blood pressure: for every 20 mmHg increase in usual systolic blood pressure (SBP) or 10 mmHg increase in usual diastolic blood pressure (DBP), stroke death rates double (23,24). Lowering blood pressure in randomized trials is associated with a comparable risk reduction, confirming that this relationship is causal (25).

The results from multiple studies in dialysis patients are far less compelling. At least three studies have found no independent association between stroke risk and measures of blood pressure (21,26,27), while two reasonably large studies have found only weakly positive associations (20,28). In unadjusted analyses from a study of 8920 US dialysis patients, *mean* predialysis blood pressure (separate systolic and diastolic results were not presented) was not associated with stroke risk (unadjusted hazard ratio [HR] 1.00, 95% CI 0.95–1.05). After adjustment for potential confounders, every 10 mmHg increment in *mean* predialysis blood pressure was associated with only an 11% increased risk of stroke (adjusted HR 1.11, 95% CI 1.05–1.18) (20).

There are several reasons why these analyses may have underestimated the true magnitude of any adverse effect of blood pressure in dialysis patients. First, measurement of blood pressure once a patient's kidney disease has progressed to require dialysis may not represent past exposure because: (a) blood pressure is modified by both dialysis and medication, and is generally treated to a guideline target (29) (80% of US dialysis patients are prescribed at least one antihypertensive drug (30)); and (b) long-standing hypertension (and other cardiovascular diseases) can lead to lower blood pressure by reducing cardiac contractility (31,32). The majority of incident dialysis patients have evidence of structural heart disease which worsens with increasing dialysis vintage (33). As hypertensive heart disease may both lower blood pressure and increase stroke risk, its presence may not only weaken the observed association between blood pressure and stroke, it may even reverse it, resulting in “J” or “U” shaped associations between blood pressure and adverse outcomes (this type of bias is referred to as reverse causality) (34,35).

Secondly, it is important to consider the various measures of blood pressure. High blood pressure in dialysis patients results from extracellular volume expansion, increased sympathetic activity, and vascular stiffness (36). Vascular stiffness reduces systolic compliance and diastolic recoil (37,38) acting to increase SBP while simultaneously lowering DBP. Diastolic and mean blood pressure readings are therefore more complicated exposures to consider in this population.

**Fig. 2:**
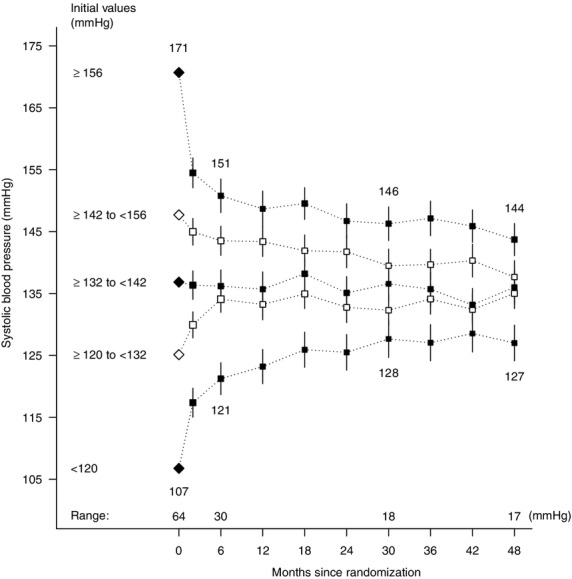
Mean systolic blood pressure over follow-up time for SHARP participants on dialysis at randomization in categories defined by quintiles of baseline measurement. SHARP, Study of Heart and Renal Protection

Thirdly, diurnal variation in blood pressure in dialysis patients can be considerable, particularly on dialysis days. Predialysis BP is significantly greater than the daily average BP (as estimated from ambulatory BP measurements), and postdialysis BP recordings are significantly lower (39). Daily blood pressure fluctuation within individuals means that a single reading may not be indicative of a patient's “usual” blood pressure, and may result in patients being miscategorized. On repeat measurement, an individual's blood pressure tends to be nearer the population mean than on first measurement. When the Study of Heart and Renal Protection (SHARP) (40) dialysis patients' SBP data were divided by quintiles based on a single baseline measurement, those in the highest quintile had an average SBP of 171 mmHg. On remeasurement at 2 months, average SBP in this group was 151 mmHg and at 30 months, (the approximate study midpoint) it was 146 mmHg. The mean SBP in the lowest quintile at baseline was 107 mmHg. By 2 months, average SBP in this group had increased to 121 mmHg and by 30 months, it was 128 mmHg (Fig. 2).

The observed regression to the mean in SHARP is typical, and if it is not taken into account by observational studies, the relevance of SBP to stroke will be systematically underestimated by such studies—the resulting bias is termed regression-dilution bias. This underestimate can be corrected by dividing the observed coefficient relating disease risk to the exposure (i.e., the natural log of the risk ratio [RR]) by its regression-dilution ratio (defined as the average difference in SBP between the highest and lowest quintiles of SBP at the study midpoint divided by the difference at baseline [i.e., 18 mmHg/64 mmHg = 0.28]) (23,41). This would mean, for the sake of argument, if the risk ratio for the association between a single measurement of SBP and stroke in an observational study of dialysis patients was 1.2 per 20 mmHg higher SBP, the relevance of “usual” SBP to stroke would be 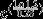, a 1.9-fold increased risk per 20 mmHg.

Similar types of bias to those described above are common in observational data from diseased populations and mean that observations from dialysis cohorts must be interpreted cautiously. In the case of blood pressure, the apparently weak association between blood pressure and stroke in those on dialysis should not be interpreted as justification for more relaxed blood pressure targets, and the optimum targets with which to treat modifiable risk factors that may be affected by reverse causality (such as blood pressure or HbA1c) should not be identified by examining the lowest point in the “J” or “U” shaped association curves.

As randomization controls for reverse causality and other biases, the true relevance of blood pressure to cardiovascular risk in dialysis patients may well be best estimated from trials. Meta-analysis of eight randomized trials of antihypertensive treatments in 1679 hemodialysis patients has demonstrated that there are benefits to lowering blood pressure in this population: a mean reduction in SBP of 4.5 mmHg and DBP of 2.3 mmHg resulted in a 29% lower risk of cardiovascular events (risk ratio [RR] 0.71, 95% CI 0.55–0.92; effect on stroke was not presented). There was no heterogeneity between those considered hypertensive and those not (42).

The optimum therapeutic blood pressure target in dialysis patients, however, remains uncertain. This question has been addressed in people with diabetes mellitus (but not with CKD) by the Action to Control Cardiovascular Risk in Diabetes (ACCORD) study, which compared a “normalized” SBP (<120 mmHg) with an intensive blood pressure lowering regimen versus a less intensive regimen. An average SBP of 119 mmHg versus 134 mmHg was achieved in the two treatment arms, resulting in a 41% reduction in the risk of stroke (HR 0.59, 95% CI 0.39–0.89) (43). A trial of comparable design in dialysis patients would help provide information about whether blood pressure should be reduced to lower levels than are currently recommended.

## Atherosclerotic Cerebrovascular Disease Prevention and Treatment

### Lowering Low-Density Lipoprotein (LDL) Cholesterol

Observational analyses among dialysis patients suggest an apparent lack of any association between higher cholesterol and mortality. Indeed, those with very low total cholesterol concentrations are at highest risk of death and there is a flat relationship at cholesterol levels within the “normal” range (44). It has been hypothesized that the presence of inflammation may explain the negative association at low total cholesterol concentrations, as inflammation both inhibits cholesterol synthesis and is associated with cardiovascular risk. The same bias may confound the relationship between cholesterol and stroke in those on dialysis, in whom no consistent association between total, non-high-density lipoprotein (non-HDL) or LDL cholesterol concentration and stroke risk has been observed (summarized in Table 1) (20).

In apparently healthy people, large-scale meta-analysis of observational studies has demonstrated a modest positive relationship between non-HDL cholesterol (made up mostly of LDL cholesterol) and ischemic stroke risk (45). Unadjusted analyses from a 45,390 Japanese haemodialysis patient registry found that neither higher non-HDL cholesterol, nor higher LDL cholesterol were associated with ischemic stroke risk until adjustment was made for predictors of protein energy wasting and inflammation. After such adjustment, the size of the association between higher non-HDL cholesterol and ischemic stroke in hemodialysis patients (HR per 39 mg/dl higher non-HDL cholesterol = 1.11, 95% CI 1.04–1.19) (18) was similar to the adjusted association observed in apparently healthy adults (HR per 43 mg/dl higher non-HDL cholesterol = 1.12, 95% CI 1.04–1.20) (45).

In a wide range of high-risk populations, reducing LDL cholesterol with statin-based regimes clearly reduces ischemic stroke risk (46). Randomized trials, by avoiding the biases inherent to observational studies, have been able to demonstrate that LDL cholesterol is a risk factor for major atherosclerotic events (including ischemic stroke) in those with advanced CKD, thereby illustrating the difficulty of relying on observational studies to guide therapeutic decisions in those with CKD (or indeed in any population). SHARP demonstrated that lowering LDL cholesterol by 33 mg/dl in 9270 CKD patients without a previous myocardial infarction (MI) or coronary revascularization reduced risk of major atherosclerotic events by 17% (RR 0.83; 95% CI 0.74–0.94). This included a significant reduction in the risk of a nonhemorrhagic stroke (RR 0.75; 95% CI 0.60–0.94). There were comparable proportional benefits for major atherosclerotic events in patients already on dialysis at baseline and those that were not (test for heterogeneity *p* = 0.25), with those on dialysis predicted to benefit more, in absolute terms, due to higher baseline risk of an atherosclerotic event (40). SHARP also demonstrated that intensive lowering of LDL cholesterol in advanced CKD is safe, therefore lowering LDL cholesterol with statin-based therapy should be considered a key part of any strategy to reduce dialysis patients' ischemic stroke and other atherosclerotic disease risk (47).

### Carotid Revascularization

Carotid atherosclerosis is a well-known risk factor for stroke in the general population and surgical removal of such plaque can reduce the risk of future cerebrovascular disease (48,49). For patients with a recent (<6 months) ischemic cerebrovascular event and 70–99% ipsilateral carotid stenosis, carotid endarterectomy is recommended when the perioperative morbidity and mortality risk is estimated to be <6% and life expectancy is sufficiently long (and may be considered for symptomatic stenosis of 50–69%) (49,50).

Carotid stenting is a less invasive alternative to endarterectomy. In the 2502 patient Carotid Revascularization Endarterectomy versus Stenting Trial (CREST), carotid stenting was associated with half the risk of a periprocedural MI, but almost twice the risk of a periprocedural stroke. As the absolute risks of a periprocedural stroke or MI were similar in the population studied, the risk of the primary outcome of stroke, MI, or death did not differ between the two treatment arms (HR 1.11, 95% CI 0.81–1.51) (51). Dialysis patients are at high risk of operative complications, including MI (52), but the heavy burden of carotid calcification also predisposes them to periprocedural stroke (53–55). It therefore remains unclear whether stenting is any safer than endarterectomy for carotid stenosis in those on dialysis (49).

## Thromboembolic Stroke Prevention

Less than one-fifth of patients commencing dialysis have a normal echocardiogram (56). Left ventricular hypertrophy is very common and often accompanied by ventricular dilatation, coronary atherosclerosis with calcification, and increased arterial stiffness. Consequently, congestive heart failure manifests in about a third of incident dialysis patients (57), and the prevalence of atrial fibrillation (AF) is high (13% of US hemodialysis patients aged 65–75 years; 19% of those aged 75–85 years; and 23% of those over 85 years have a history of AF (58)).

The Framingham study demonstrated that AF increases stroke risk 5-fold (59). In addition, reduced kidney function increases the probability of thromboembolic complications of AF (60). Correspondingly, AF in dialysis patients has been associated with significantly increased ischemic stroke risk (Table 1) (21,22,27,61,62).

There is evidence from randomized trials that anticoagulation reduces thromboembolic stroke risk in AF in patients from non-dialysis populations, and there is net benefit in those at moderate to high stroke risk (63–66). Meta-analysis of the large trials of the direct thrombin inhibitor dabigatran and the factor Xa inhibitors (rivaroxaban, apixaban, and edoxaban) has demonstrated that these newer agents are as effective as warfarin at preventing ischemic stroke (RR 0.92, 95% CI 0.83–1.02) and are less likely to cause a hemorrhagic stroke (RR 0.47, 95% CI 0.38–0.64) (67). All these newer anticoagulants are eliminated (at least in part) by the kidneys, but few patients with advanced CKD were included in the definitive trials. Currently, none of these agents have a license for use in dialysis patients (68). Anxieties about the safety of warfarin in those on dialysis also exist, not only because pretreatment bleeding risk is already high (69), but also because inhibition of vitamin K-dependent carboxylation of matrix Gla protein (a calcification inhibitor) raises the possibility that warfarin may accelerate arterial calcification and thereby increase the risk of non-cardioembolic ischemic stroke (and other vascular disease) (62,70–74). This has led some to recommend that dialysis patients with non-valvular AF should *not* routinely be considered for thromboprophylaxis (70), but there is no large-scale randomized evidence in dialysis to support this view (Table 2).

**Table 2:** Key areas of uncertainty surrounding the efficacy and safety of strategies to prevent and treat stroke in dialysis patients

All stroke
Optimal targets for blood pressure control
Frequency and duration of hemodialysis
Phosphate reduction using calcium- and/or non-calcium-based binders
Suppression of parathyroid hormone with calcimimetics or vitamin D therapy
Ischemic stroke
Aspirin (or other antiplatelet agents) for primary and secondary prevention
Thrombolysis for acute ischemic stroke
Carotid artery endarterectomy or stenting for treatment of large vessel cerebrovascular disease
Thromboprophylaxis with warfarin (or novel anticoagulants) for atrial fibrillation
Hemorrhagic stroke
Regional anticoagulation compared to systemic anticoagulation for hemodialysis

Five recent observational analyses of the association between warfarin prescription and stroke in those on dialysis with AF have yielded conflicting results (perhaps reflecting different amounts of residual confounding between these studies (75)): one study in US dialysis patients with AF found warfarin prescription to be associated with an increased risk of ischemic stroke (72), in two other North American studies, there was no significant association (73,76), while two from Scandinavia suggested that warfarin prescription in those on renal replacement therapy (or with advanced CKD) was associated with reduced risk of stroke (or systemic thromboembolism) (69,77).

## Antiplatelet Therapy

Among patients at high risk of occlusive vascular disease, the Antithrombotic Trialists' (ATT) collaboration meta-analysis of randomized trials has shown that aspirin reduces the risk of ischemic stroke risk by about one-fifth (RR 0.83, 95% CI 0.73–0.95). Aspirin also increased the risk of a hemorrhagic stroke by about two-fifths (RR 1.39, 95% CI 1.08–1.78) (78). In the Western populations studied in these trials, because ischemic stroke is much more common than hemorrhagic stroke, the net effect on first stroke of any type was an overall reduction in risk (RR 0.89, 95% CI 0.82–0.97). Because hemorrhagic strokes have a worse outcome than ischemic strokes, the net relative effect on fatal stroke would be expected to be somewhat smaller. When attention was restricted to fatal strokes, there was no significant effect on any such strokes (RR 1.15, 95% CI 0.94–1.41) (78).

The best evidence for the effect of antiplatelet therapy on vascular risk in dialysis patients come from randomized trials primarily designed to investigate the effects of antithrombotic therapy on vascular access patency. In a subgroup analysis by the ATT meta-analysis, aspirin was found to reduce serious vascular events (nonfatal MI, nonfatal stroke, or vascular death) in hemodialysis patients by 41% (RR 0.59, 95% CI 0.40–0.89) (79).

The ATT also demonstrated that aspirin clearly increases the risk of major extracranial bleeding. For the average trial patient with established vascular disease (i.e., secondary prevention), the absolute benefit of antiplatelet therapy was an order of magnitude greater than the risk of harm (78). However, the net effect of antiplatelet therapy in dialysis patients, who are at substantially increased risk of serious bleeding (69), has not been tested in an adequately sized trial. Based on event rates among dialysis patients in SHARP (and applying the summary risk ratios for aspirin from the ATT meta-analysis (78)), treating 1000 dialysis patients *with* vascular disease with aspirin for 5 years is projected to prevent 16 ischemic strokes and 75 MIs or revascularizations, but also cause an additional 19 intracranial bleeds and 53 serious extracranial bleeds. These estimates raise considerable uncertainty and emphasize the need for a large randomized trial of antiplatelet therapy in dialysis patients both with and without established vascular disease (Table 2).

## Acute Stroke Treatment

Among patients without CKD, individual patient data meta-analysis by the Stroke Thrombolysis Trialists' (STT) collaboration including 6756 patients from nine randomized trials has demonstrated that, despite an early hazard of symptomatic intracranial hemorrhage, intravenous recombinant tissue plasminogen activator (rt-PA) administered within 4.5 hours of the onset of symptoms clearly increased disability-free survival at 3–6 months (80). The proportional benefits were greater if rt-PA was administered within 3 hours.

However, there was a clear early hazard of fatal intracranial hemorrhage within 7 days (OR 7.14, 95% CI 3.98–12.79). In absolute terms, this equated to an increased risk of early death due to intracranial hemorrhage of about 2%, in exchange for an increase in disability-free survival of about 10% for treatment within 3 hours and about 5% for those treated between 3 and 4.5 hours (80). As advanced CKD is associated with 3-fold increased risk of hemorrhagic transformation after ischemic stroke (odds ratio 2.90, 95% CI 1.26–6.68) (81), should the proportional effects of rt-PA observed in the STT meta-analysis remain the same for those on dialysis, the absolute risk of early death due to intracranial hemorrhage from rt-PA might similarly increase 3-fold (i.e., to about 6%) and outweigh the potential benefit. When surveyed, two-thirds of nephrologists in the United Kingdom have a high degree of concern about thrombolysis (82). Uncertainty has also been identified in a survey of US stroke specialists, who prefer the use of mechanical clot retrieval over intravenous rt-PA in dialysis patients (Table 2) (83). Whether or not a dialysis patient with an ischemic stroke receives rt-PA, it is important that regular dialysis does not then interfere with the proven benefit of rehabilitation in stroke units (84).

## Effects of Renal-Specific Therapies on Stroke Risk

### Treatments for Mineral and Bone Disorder

Arterial medial calcification of intracranial vessels becomes increasingly prevalent with advanced age and reduced renal function (85). Calcification results in increased vessel wall thickness and stiffness, which increases SBP and pulse-wave velocity (both of which are associated with increased risk of stroke (23,17) and stroke death (37,38)). Mineral and bone disorder may contribute to accelerated vascular calcification (86), and its markers (increased serum phosphate, calcium, parathyroid hormone [PTH], and fibroblast growth factor 23 concentration) have all been independently associated with increased mortality in dialysis patients (87–89). However, a causal role for mineral and bone disorder in cardiovascular disease (including stroke) has yet to be demonstrated. Randomized trials of phosphate binders have not been sufficiently powered to test the effect of this phosphate reduction on hard cardiovascular outcomes (90) and lowering PTH by about 250 pg/ml and calcium concentration by about 0.4 mg/dl in the 3883 dialysis patient Evaluation of Cinacalcet Hydrochloride Therapy to Lower Cardiovascular Events (EVOLVE) trial had no significant effect on the primary outcome of death, MI, hospitalization for unstable angina, heart failure, or a peripheral vascular event (HR 0.93, 95% CI 0.85–1.02; information on the separate effect on stroke was not presented) (91).

### Hemodialysis

Data from the United States Renal Data Service (USRDS) suggest there is a greater than 4-fold increase in the risk of stroke in the first month after starting dialysis (10,92). A question was therefore raised as to whether the dialysis process is a “stress test” and is responsible for some of the increased risk of stroke in dialysis patients, perhaps by causing reduced cerebral perfusion during ultrafiltration or episodes of intradialytic hypotension, or by increasing the risk of hemorrhagic stroke by use of anticoagulation (despite dialysis also abrogating uremic bleeding risk).

When the timing of strokes is examined, there does appear to be an increase in stroke presentation around the time of a dialysis session. Most hemodialysis patients spend about 7% of their week (12 hours a week) on dialysis. Among the 58 hemodialysis patients with a hemorrhagic stroke in a Japanese study, 10% occurred during hemodialysis with a further 9% shortly afterward (93). Of the 86 ischemic strokes in the same study, 19% occurred during dialysis and 15% shortly after (93). Of the 90, 11% ischemic strokes in the US hemodialysis patients from the CHOICE study occurred during hemodialysis (21). Although these data suggest the dialysis process itself may indeed cause some strokes, a much large observational study of hospital admissions in US hemodialysis patients identified that the peak day of the week for a dialysis patient to suffer a stroke is on the dialysis day after the long interdialytic period, when metabolic and volume control is at its worst (94). One might therefore hypothesize that more frequent or longer dialysis may be an important intervention to prevent stroke. Randomized studies of daily (nocturnal) hemodialysis found increased dialysis frequency led to a fall in blood pressure, reduced left ventricular mass, and improved markers of mineral and bone disorder, all of which might theoretically reduce stroke risk (95,96).

Refinements to the hemodialysis process may also be helpful in reducing stroke risk. The addition of convection therapy promotes cardiovascular stability. In the randomized Online Hemodiafiltration (HDF) Survival Study, HDF reduced episodes of intradialytic hypotension by 28% (RR 0.72, 95% CI 0.68–0.77) compared to thrice weekly hemodialysis. HDF also reduced all-cause mortality by 30% (RR 0.70, 95% CI 0.53–0.92), which included fewer stroke deaths (RR 0.39, 95% CI 0.16–0.93; the effect on nonfatal stroke was not presented (97)).

### Vascular Access

Vascular access may increase stroke risk through multiple mechanisms. First, data from a period when antibacterial catheter locks were less widely used found about one in 10 strokes in dialysis patients were related to endocarditis (98). Secondly, a stroke at the time of a vascular access procedure should prompt investigation for a persistent patent foramen ovale (99). Lastly, vascular access may also affect cerebral hemodynamics: a Japanese hospital stroke registry examined 1168 new strokes including 151 among maintenance hemodialysis (93,100). Of the 86 ischemic strokes in hemodialysis patients, vertebrobasilar disease made up 43% of strokes, compared to 33% of those not on dialysis (93). The authors raised the hypothesis (that clearly needs further testing) that arteriovenous vascular access may account for this possible difference, perhaps by altering flow velocity of the vertebral artery as a result of low shunt resistance.

### Erythropoiesis-Stimulating Agents

Anemia has been associated with stroke risk in CKD (101). This observation may simply be due to confounding by ill health, as inflammation reduces the erythropoietin response. It has also been hypothesized that anemia may promote structural heart disease such as left ventricular hypertrophy, thereby increasing stroke risk (102,103). However, the relationship between anemia and left ventricular hypertrophy may not be causal, as correction of anemia in CKD in the Cardiovascular Risk Reduction by Early Anemia Treatment with Epoetin Beta (CREATE) trial did not affect left ventricular mass (104). Moreover, the relationship between stroke and anemia is reversed in dialysis patients, with low or normal hemoglobin (Hb) appearing protective (92,103). The Trial to Reduce Cardiovascular Events with Aranesp Therapy (TREAT) studied diabetic patients with CKD not on dialysis, and found a clear doubling of stroke risk in the higher Hb target arm (target Hb 13 vs. 9 g/dl, HR 1.92, 95% CI 1.38–2.68) (19), a hazard that was not modified by any baseline characteristic. An excess of venous thromboembolic complications (41 [2.0%] vs. 23 [1.1%]) was also observed in the higher Hb target group. However, the observation that the stroke hazard appeared to double for ischemic and hemorrhagic strokes considered separately suggests that, in addition to prothrombotic effects, erythropoiesis-stimulating agents are causing stroke through additional mechanisms, of which the 2 mmHg higher DBP in the higher Hb target group remains the most plausible (105).

The Normal Hematocrit study randomized 1233 dialysis patients to a “normal” hematocrit (0.42) versus a low-target hematocrit (0.30). The study was terminated early when the risk ratio for the primary endpoint (all-cause mortality and nonfatal MI) for the “normal” hematocrit group was 1.3 (95% CI 0.9–1.9) (106). There was also a non-significant excess of stroke deaths (14 [7.2%] “normal” hematocrit vs. 9 [5.6%] low hematocrit; data on nonfatal strokes have not been published (107)). These results highlight the need for large-scale trials of widely used current treatments, as well as novel ones.

## Summary

The risk of both ischemic and hemorrhagic stroke is particularly high in dialysis patients of all ages, but there is a lack of reliable evidence in dialysis patients on which to recommend interventions for the prevention of stroke or for its acute treatment. Observational studies in dialysis patients are particularly affected by biases that usually make them an inappropriate source of data on which to base therapeutic decisions. Consequently, it is important that reliable information on the prevention and treatment of stroke (and other cardiovascular disease) in dialysis patients is generated by performing more large-scale randomized trials of many current and future treatments.

## References

[1] Liu M, Wu B, Wang WZ, Lee LM, Zhang SH, Kong LZ (2007). Stroke in China: epidemiology, prevention, and management strategies. Lancet Neurol.

[2] Thorvaldsen P, Asplund K, Kuulasmaa K, Rajakangas AM, Schroll M (1995). Stroke incidence, case fatality, and mortality in the WHO MONICA project. World Health Organization Monitoring Trends and Determinants in Cardiovascular Disease. Stroke.

[3] Lozano R, Naghavi M, Foreman K, Lim S, Shibuya K, Aboyans V, Abraham J, Adair T, Aggarwal R, Ahn SY, Alvarado M, Anderson HR, Anderson LM, Andrews KG, Atkinson C, Baddour LM, Barker-Collo S, Bartels DH, Bell ML, Benjamin EJ, Bennett D, Bhalla K, Bikbov B, Bin AA, Birbeck G, Blyth F, Bolliger I, Boufous S, Bucello C, Burch M, Burney P, Carapetis J, Chen H, Chou D, Chugh SS, Coffeng LE, Colan SD, Colquhoun S, Colson KE, Condon J, Connor MD, Cooper LT, Corriere M, Cortinovis M, de Vaccaro KC, Couser W, Cowie BC, Criqui MH, Cross M, Dabhadkar KC, Dahodwala N, De LD, Degenhardt L, Delossantos A, Denenberg J, Des Jarlais DC, Dharmaratne SD, Dorsey ER, Driscoll T, Duber H, Ebel B, Erwin PJ, Espindola P, Ezzati M, Feigin V, Flaxman AD, Forouzanfar MH, Fowkes FG, Franklin R, Fransen M, Freeman MK, Gabriel SE, Gakidou E, Gaspari F, Gillum RF, Gonzalez-Medina D, Halasa YA, Haring D, Harrison JE, Havmoeller R, Hay RJ, Hoen B, Hotez PJ, Hoy D, Jacobsen KH, James SL, Jasrasaria R, Jayaraman S, Johns N, Karthikeyan G, Kassebaum N, Keren A, Khoo JP, Knowlton LM, Kobusingye O, Koranteng A, Krishnamurthi R, Lipnick M, Lipshultz SE, Ohno SL, Mabweijano J, MacIntyre MF, Mallinger L, March L, Marks GB, Marks R, Matsumori A, Matzopoulos R, Mayosi BM, McAnulty JH, McDermott MM, McGrath J, Mensah GA, Merriman TR, Michaud C, Miller M, Miller TR, Mock C, Mocumbi AO, Mokdad AA, Moran A, Mulholland K, Nair MN, Naldi L, Narayan KM, Nasseri K, Norman P, O'Donnell M, Omer SB, Ortblad K, Osborne R, Ozgediz D, Pahari B, Pandian JD, Rivero AP, Padilla RP, Perez-Ruiz F, Perico N, Phillips D, Pierce K, Pope CA, Porrini E, Pourmalek F, Raju M, Ranganathan D, Rehm JT, Rein DB, Remuzzi G, Rivara FP, Roberts T, De León FR, Rosenfeld LC, Rushton L, Sacco RL, Salomon JA, Sampson U, Sanman E, Schwebel DC, Segui-Gomez M, Shepard DS, Singh D, Singleton J, Sliwa K, Smith E, Steer A, Taylor JA, Thomas B, Tleyjeh IM, Towbin JA, Truelsen T, Undurraga EA, Venketasubramanian N, Vijayakumar L, Vos T, Wagner GR, Wang M, Wang W, Watt K, Weinstock MA, Weintraub R, Wilkinson JD, Woolf AD, Wulf S, Yeh PH, Yip P, Zabetian A, Zheng ZJ, Lopez AD, Murray CJ, AlMazroa MA, Memish ZA (2012). Global and regional mortality from 235 causes of death for 20 age groups in 1990 and 2010: a systematic analysis for the Global Burden of Disease Study 2010. Lancet.

[4] Murray CJ, Vos T, Lozano R, Naghavi M, Flaxman AD, Michaud C, Ezzati M, Shibuya K, Salomon JA, Abdalla S, Aboyans V, Abraham J, Ackerman I, Aggarwal R, Ahn SY, Ali MK, Alvarado M, Anderson HR, Anderson LM, Andrews KG, Atkinson C, Baddour LM, Bahalim AN, Barker-Collo S, Barrero LH, Bartels DH, Basáñez MG, Baxter A, Bell ML, Benjamin EJ, Bennett D, Bernabé E, Bhalla K, Bhandari B, Bikbov B, Bin Abdulhak A, Birbeck G, Black JA, Blencowe H, Blore JD, Blyth F, Bolliger I, Bonaventure A, Boufous S, Bourne R, Boussinesq M, Braithwaite T, Brayne C, Bridgett L, Brooker S, Brooks P, Brugha TS, Bryan-Hancock C, Bucello C, Buchbinder R, Buckle G, Budke CM, Burch M, Burney P, Burstein R, Calabria B, Campbell B, Canter CE, Carabin H, Carapetis J, Carmona L, Cella C, Charlson F, Chen H, Cheng AT, Chou D, Chugh SS, Coffeng LE, Colan SD, Colquhoun S, Colson KE, Condon J, Connor MD, Cooper LT, Corriere M, Cortinovis M, de Vaccaro KC, Couser W, Cowie BC, Criqui MH, Cross M, Dabhadkar KC, Dahiya M, Dahodwala N, Damsere-Derry J, Danaei G, Davis A, De Leo D, Degenhardt L, Dellavalle R, Delossantos A, Denenberg J, Derrett S, Des Jarlais DC, Dharmaratne SD, Dherani M, Diaz-Torne C, Dolk H, Dorsey ER, Driscoll T, Duber H, Ebel B, Edmond K, Elbaz A, Ali SE, Erskine H, Erwin PJ, Espindola P, Ewoigbokhan SE, Farzadfar F, Feigin V, Felson DT, Ferrari A, Ferri CP, Fèvre EM, Finucane MM, Flaxman S, Flood L, Foreman K, Forouzanfar MH, Fowkes FG, Fransen M, Freeman MK, Gabbe BJ, Gabriel SE, Gakidou E, Ganatra HA, Garcia B, Gaspari F, Gillum RF, Gmel G, Gonzalez-Medina D, Gosselin R, Grainger R, Grant B, Groeger J, Guillemin F, Gunnell D, Gupta R, Haagsma J, Hagan H, Halasa YA, Hall W, Haring D, Haro JM, Harrison JE, Havmoeller R, Hay RJ, Higashi H, Hill C, Hoen B, Hoffman H, Hotez PJ, Hoy D, Huang JJ, Ibeanusi SE, Jacobsen KH, James SL, Jarvis D, Jasrasaria R, Jayaraman S, Johns N, Jonas JB, Karthikeyan G, Kassebaum N, Kawakami N, Keren A, Khoo JP, King CH, Knowlton LM, Kobusingye O, Koranteng A, Krishnamurthi R, Laden F, Lalloo R, Laslett LL, Lathlean T, Leasher JL, Lee YY, Leigh J, Levinson D, Lim SS, Limb E, Lin JK, Lipnick M, Lipshultz SE, Liu W, Loane M, Ohno SL, Lyons R, Mabweijano J, MacIntyre MF, Malekzadeh R, Mallinger L, Manivannan S, Marcenes W, March L, Margolis DJ, Marks GB, Marks R, Matsumori A, Matzopoulos R, Mayosi BM, McAnulty JH, McDermott MM, McGill N, McGrath J, Medina-Mora ME, Meltzer M, Mensah GA, Merriman TR, Meyer AC, Miglioli V, Miller M, Miller TR, Mitchell PB, Mock C, Mocumbi AO, Moffitt TE, Mokdad AA, Monasta L, Montico M, Moradi-Lakeh M, Moran A, Morawska L, Mori R, Murdoch ME, Mwaniki MK, Naidoo K, Nair MN, Naldi L, Narayan KM, Nelson PK, Nelson RG, Nevitt MC, Newton CR, Nolte S, Norman P, Norman R, O'Donnell M, O'Hanlon S, Olives C, Omer SB, Ortblad K, Osborne R, Ozgediz D, Page A, Pahari B, Pandian JD, Rivero AP, Patten SB, Pearce N, Padilla RP, Perez-Ruiz F, Perico N, Pesudovs K, Phillips D, Phillips MR, Pierce K, Pion S, Polanczyk GV, Polinder S, Pope CA, Popova S, Porrini E, Pourmalek F, Prince M, Pullan RL, Ramaiah KD, Ranganathan D, Razavi H, Regan M, Rehm JT, Rein DB, Remuzzi G, Richardson K, Rivara FP, Roberts T, Robinson C, De LF, Ronfani L, Room R, Rosenfeld LC, Rushton L, Sacco RL, Saha S, Sampson U, Sanchez-Riera L, Sanman E, Schwebel DC, Scott JG, Segui-Gomez M, Shahraz S, Shepard DS, Shin H, Shivakoti R, Singh D, Singh GM, Singh JA, Singleton J, Sleet DA, Sliwa K, Smith E, Smith JL, Stapelberg NJ, Steer A, Steiner T, Stolk WA, Stovner LJ, Sudfeld C, Syed S, Tamburlini G, Tavakkoli M, Taylor HR, Taylor JA, Taylor WJ, Thomas B, Thomson WM, Thurston GD, Tleyjeh IM, Tonelli M, Towbin JA, Truelsen T, Tsilimbaris MK, Ubeda C, Undurraga EA, van der Werf MJ, van Os J, Vavilala MS, Venketasubramanian N, Wang M, Wang W, Watt K, Weatherall DJ, Weinstock MA, Weintraub R, Weisskopf MG, Weissman MM, White RA, Whiteford H, Wiebe N, Wiersma ST, Wilkinson JD, Williams HC, Williams SR, Witt E, W:olfe F, Woolf AD, Wulf S, Yeh PH, Zaidi AK, Zheng ZJ, Zonies D, Lopez AD, AlMazroa MA, Memish ZA (2012). Disability-adjusted life years (DALYs) for 291 diseases and injuries in 21 regions, 1990-2010: a systematic analysis for the Global Burden of Disease Study 2010. Lancet.

[5] Lloyd-Jones D, Adams RJ, Brown TM, Carnethon M, Dai S, De Simone G, Ferguson TB, Ford E, Furie K, Gillespie C, Go A, Greenlund K, Haase N, Hailpern S, Ho PM, Howard V, Kissela B, Kittner S, Lackland D, Lisabeth L, Marelli A, McDermott MM, Meigs J, Mozaffarian D, Mussolino M, Nichol G, Roger VL, Rosamond W, Sacco R, Sorlie P, Stafford R, Thom T, Wasserthiel-Smoller S, Wong ND, Wylie-Rosett J, WG (2010). Heart disease and stroke statistics–2010 update: a report from the American Heart Association. Circulation.

[6] Adams HP, Bendixen BH, Kappelle LJ, Biller J, Love BB, Gordon DL, Marsh EE (1993). Classification of subtype of acute ischemic stroke. Definitions for use in a multicenter clinical trial. TOAST. Trial of Org 10172 in Acute Stroke Treatment. Stroke.

[7] Jackson CA, Sudlow CL (2006). Is hypertension a more frequent risk factor for deep than for lobar supratentorial intracerebral haemorrhage?. J Neurol Neurosurg Psychiatry.

[8] van Gijn J, Rinkel GJ (2001). Subarachnoid haemorrhage: diagnosis, causes and management. Brain.

[9] Go AS, Chertow GM, Fan D, McCulloch CE, Hsu CY (2004). Chronic kidney disease and the risks of death, cardiovascular events, and hospitalization. N Engl J Med.

[10] U.S. Renal Data System (2009). USRDS 2009 Annual Data Report: Atlas of Chronic Kidney Disease and End-Stage Renal Disease in the United States.

[11] Seliger SL, Gillen DL, Longstreth WT, Kestenbaum B, Stehman-Breen CO (2003). Elevated risk of stroke among patients with end-stage renal disease. Kidney Int.

[12] Wang HH, Hung SY, Sung JM, Hung KY, Wang JD (2014). Risk of stroke in long-term dialysis patients compared with the general population. Am J Kidney Dis.

[13] Ovbiagele B (2011). Chronic kidney disease and risk of death during hospitalization for stroke. J Neurol Sci.

[14] U.S. Renal Data System (2011). USRDS 2010 Annual Data Report: Atlas of Chronic Kidney Disease and End-Stage Renal Disease in the United States.

[15] Sarwar N, Gao P, Seshasai SR, Gobin R, Kaptoge S, Di Angelantonio E, Ingelsson E, Lawlor DA, Selvin E, Stampfer M, Stehouwer CD, Lewington S, Pennells L, Thompson A, Sattar N, White IR, Ray KK, Danesh J (2010). Diabetes mellitus, fasting blood glucose concentration, and risk of vascular disease: a collaborative meta-analysis of 102 prospective studies. Lancet.

[16] London GM, Guérin AP, Marchais SJ, Métivier F, Pannier B, Adda H (2003). Arterial media calcification in end-stage renal disease: impact on all-cause and cardiovascular mortality. Nephrol Dial Transplant.

[17] Sutton-Tyrrell K, Najjar SS, Boudreau RM, Venkitachalam L, Kupelian V, Simonsick EM, Havlik R, Lakatta EG, Spurgeon H, Kritchevsky S, Pahor M, Bauer D, Newman A (2005). Elevated aortic pulse wave velocity, a marker of arterial stiffness, predicts cardiovascular events in well-functioning older adults. Circulation.

[18] Shoji T, Masakane I, Watanabe Y, Iseki K, Tsubakihara Y (2011). Committee of Renal Data Registry JpSfDT. Elevated non-high-density lipoprotein cholesterol (non-HDL-C) predicts atherosclerotic cardiovascular events in hemodialysis patients. Clin J Am Soc Nephrol.

[19] Pfeffer MA, Burdmann EA, Chen CY, Cooper ME, de Zeeuw D, Eckardt KU, Feyzi JM, Ivanovich P, Kewalramani R, Levey AS, Lewis EF, McGill JB, McMurray JJ, Parfrey P, Parving HH, Remuzzi G, Singh AK, Solomon SD, Toto R (2009). A trial of darbepoetin alfa in type 2 diabetes and chronic kidney disease. N Engl J Med.

[20] Seliger SL, Gillen DL, Tirschwell D, Wasse H, Kestenbaum BR, Stehman-Breen CO (2003). Risk factors for incident stroke among patients with end-stage renal disease. J Am Soc Nephrol.

[21] Sozio SM, Armstrong PA, Coresh J, Jaar BG, Fink NE, Plantinga LC, Powe NR, Parekh RS (2009). Cerebrovascular disease incidence, characteristics, and outcomes in patients initiating dialysis: the choices for healthy outcomes in caring for ESRD (CHOICE) study. Am J Kidney Dis.

[22] Power A, Chan K, Singh SK, Taube D, Duncan N (2012). Appraising stroke risk in maintenance hemodialysis patients: a large single-center cohort study. Am J Kidney Dis.

[23] Lewington S, Clarke R, Qizilbash N, Peto R, Collins R, Prospective Studies Collaboration (2002). Age-specific relevance of usual blood pressure to vascular mortality: a meta-analysis of individual data for one million adults in 61 prospective studies. Lancet.

[24] Lawes CM, Rodgers A, Bennett DA, Parag V, Suh I, Ueshima H, MacMahon S (2003). Blood pressure and cardiovascular disease in the Asia Pacific region. J Hypertens.

[25] Law MR, Morris JK, Wald NJ (2009). Use of blood pressure lowering drugs in the prevention of cardiovascular disease: meta-analysis of 147 randomised trials in the context of expectations from prospective epidemiological studies. BMJ.

[26] Delmez JA, Yan G, Bailey J, Beck GJ, Beddhu S, Cheung AK, Kaysen GA, Levey AS, Sarnak MJ, Schwab SJ (2006). Cerebrovascular disease in maintenance hemodialysis patients: results of the HEMO Study. Am J Kidney Dis.

[27] Sánchez-Perales C, Vázquez E, García-Cortés MJ, Borrego J, Polaina M, Gutiérrez CP, Lozano C, Liébana A (2010). Ischaemic stroke in incident dialysis patients. Nephrol Dial Transplant.

[28] Iseki K, Fukiyama K (1996). Predictors of stroke in patients receiving chronic hemodialysis. Kidney Int.

[29] Holt R, Goldsmith D http://www.renal.org/Clinical/GuidelinesSection/CardiovascularDiseaseInCKD.aspx.

[30] Sozio SM, Coresh J, Jaar BG, Fink NE, Plantinga LC, Armstrong PA, Longenecker JC, Sharrett AR, Powe NR, Parekh RS (2011). Inflammatory markers and risk of cerebrovascular events in patients initiating dialysis. Clin J Am Soc Nephrol.

[31] Lip GY, Felmeden DC, Li-Saw-Hee FL, Beevers DG (2000). Hypertensive heart disease. A complex syndrome or a hypertensive ‘cardiomyopathy’?. Eur Heart J.

[32] Drazner MH (2011). The progression of hypertensive heart disease. Circulation.

[33] Foley RN, Curtis BM, Randell EW, Parfrey PS (2010). Left ventricular hypertrophy in new hemodialysis patients without symptomatic cardiac disease. Clin J Am Soc Nephrol.

[34] Zager PG, Nikolic J, Brown RH, Campbell MA, Hunt WC, Peterson D, Van Stone J, Levey A, Meyer KB, Klag MJ, Johnson HK, Clark E, Sadler JH, Teredesai P (1998). “U” curve association of blood pressure and mortality in hemodialysis patients. Medical Directors of Dialysis Clinic, Inc. Kidney Int.

[35] Port FK, Hulbert-Shearon TE, Wolfe RA, Bloembergen WE, Golper TA, Agodoa LY, Young EW (1999). Predialysis blood pressure and mortality risk in a national sample of maintenance hemodialysis patients. Am J Kidney Dis.

[36] Hörl MP, Hörl WH (2002). Hemodialysis-associated hypertension: pathophysiology and therapy. Am J Kidney Dis.

[37] Guérin AP, Pannier B, Métivier F, Marchais SJ, London GM (2008). Assessment and significance of arterial stiffness in patients with chronic kidney disease. Curr Opin Nephrol Hypertens.

[38] London GM (2005). Arteriosclerosis and arterial calcifications in chronic kidney insufficiency. Nephrol Ther.

[39] Agarwal R, Peixoto AJ, Santos SF, Zoccali C (2006). Pre- and postdialysis blood pressures are imprecise estimates of interdialytic ambulatory blood pressure. Clin J Am Soc Nephrol.

[40] Baigent C, Landray MJ, Reith C, Emberson J, Wheeler DC, Tomson C, Wanner C, Krane V, Cass A, Craig J, Neal B, Jiang L, Hooi LS, Levin A, Agodoa L, Gaziano M, Kasiske B, Walker R, Massy ZA, Feldt-Rasmussen B, Krairittichai U, Ophascharoensuk V, Fellström B, Holdaas H, Tesar V, Wiecek A, Grobbee D, de Zeeuw D, Grönhagen-Riska C, Dasgupta T, Lewis D, Herrington W, Mafham M, Majoni W, Wallendszus K, Grimm R, Pedersen T, Tobert J, Armitage J, Baxter A, Bray C, Chen Y, Chen Z, Hill M, Knott C, Parish S, Simpson D, Sleight P, Young A, Collins R (2011). The effects of lowering LDL cholesterol with simvastatin plus ezetimibe in patients with chronic kidney disease (Study of Heart and Renal Protection): a randomised placebo-controlled trial. Lancet.

[41] Clarke R, Shipley M, Lewington S, Youngman L, Collins R, Marmot M, Peto R (1999). Underestimation of risk associations due to regression dilution in long-term follow-up of prospective studies. Am J Epidemiol.

[42] Heerspink HJ, Ninomiya T, Zoungas S, de Zeeuw D, Grobbee DE, Jardine MJ, Gallagher M, Roberts MA, Cass A, Neal B, Perkovic V (2009). Effect of lowering blood pressure on cardiovascular events and mortality in patients on dialysis: a systematic review and meta-analysis of randomised controlled trials. Lancet.

[43] Cushman WC, Evans GW, Byington RP, Goff DC, Grimm RH, Cutler JA, Simons-Morton DG, Basile JN, Corson MA, Probstfield JL, Katz L, Peterson KA, Friedewald WT, Buse JB, Bigger JT, Gerstein HC, Ismail-Beigi F (2010). Effects of intensive blood-pressure control in type 2 diabetes mellitus. N Engl J Med.

[44] Lowrie EG, Lew NL (1990). Death risk in hemodialysis patients: the predictive value of commonly measured variables and an evaluation of death rate differences between facilities. Am J Kidney Dis.

[45] Di Angelantonio E, Sarwar N, Perry P, Kaptoge S, Ray KK, Thompson A, Wood AM, Lewington S, Sattar N, Packard CJ, Collins R, Thompson SG, Danesh J (2009). Major lipids, apolipoproteins, and risk of vascular disease. JAMA.

[46] Baigent C, Blackwell L, Emberson J, Holland LE, Reith C, Bhala N, Peto R, Barnes EH, Keech A, Simes J, Collins R (2010). Efficacy and safety of more intensive lowering of LDL cholesterol: a meta-analysis of data from 170,000 participants in 26 randomised trials. Lancet.

[47] Green D, Ritchie JP, Kalra PA (2014). Meta-analysis of lipid-lowering therapy in maintenance dialysis patients. Nephron Clin Pract.

[48] Rothwell PM (2008). Endarterectomy for symptomatic and asymptomatic carotid stenosis. Neurol Clin.

[49] Furie KL, Kasner SE, Adams RJ, Albers GW, Bush RL, Fagan SC, Halperin JL, Johnston SC, Katzan I, Kernan WN, Mitchell PH, Ovbiagele B, Palesch YY, Sacco RL, Schwamm LH, Wassertheil-Smoller S, Turan TN, Wentworth D (2011). Guidelines for the prevention of stroke in patients with stroke or transient ischemic attack: a guideline for healthcare professionals from the american heart association/american stroke association. Stroke.

[50] Rothwell PM, Eliasziw M, Gutnikov SA, Fox AJ, Taylor DW, Mayberg MR, Warlow CP, Barnett HJ (2003). Analysis of pooled data from the randomised controlled trials of endarterectomy for symptomatic carotid stenosis. Lancet.

[51] Brott TG, Hobson RW, Howard G, Roubin GS, Clark WM, Brooks W, Mackey A, Hill MD, Leimgruber PP, Sheffet AJ, Howard VJ, Moore WS, Voeks JH, Hopkins LN, Cutlip DE, Cohen DJ, Popma JJ, Ferguson RD, Cohen SN, Blackshear JL, Silver FL, Mohr JP, Lal BK, Meschia JF (2010). Stenting versus endarterectomy for treatment of carotid-artery stenosis. N Engl J Med.

[52] Stoner MC, Abbott WM, Wong DR, Hua HT, Lamuraglia GM, Kwolek CJ, Watkins MT, Agnihotri AK, Henderson WG, Khuri S, Cambria RP (2006). Defining the high-risk patient for carotid endarterectomy: an analysis of the prospective National Surgical Quality Improvement Program database. J Vasc Surg.

[53] Kawagishi T, Nishizawa Y, Konishi T, Kawasaki K, Emoto M, Shoji T, Tabata T, Inoue T, Morii H (1995). High-resolution B-mode ultrasonography in evaluation of atherosclerosis in uremia. Kidney Int.

[54] Zoungas S, Ristevski S, Lightfoot P, Liang YL, Branley P, Shiel LM, Kerr P, Atkins R, McNeil JJ, McGrath BP (2000). Carotid artery intima-medial thickness is increased in chronic renal failure. Clin Exp Pharmacol Physiol.

[55] Savage T, Clarke AL, Giles M, Tomson CR, Raine AE (1998). Calcified plaque is common in the carotid and femoral arteries of dialysis patients without clinical vascular disease. Nephrol Dial Transplant.

[56] Foley RN, Parfrey PS, Harnett JD, Kent GM, Martin CJ, Murray DC, Barre PE (1995). Clinical and echocardiographic disease in patients starting end-stage renal disease therapy. Kidney Int.

[57] Baigent C, Burbury K, Wheeler D (2000). Premature cardiovascular disease in chronic renal failure. Lancet.

[58] Winkelmayer WC, Patrick AR, Liu J, Brookhart MA, Setoguchi S (2011). The increasing prevalence of atrial fibrillation among hemodialysis patients. J Am Soc Nephrol.

[59] Wolf PA, Abbott RD, Kannel WB (1991). Atrial fibrillation as an independent risk factor for stroke: the Framingham Study. Stroke.

[60] Go AS, Fang MC, Udaltsova N, Chang Y, Pomernacki NK, Borowsky L, Singer DE (2009). Impact of proteinuria and glomerular filtration rate on risk of thromboembolism in atrial fibrillation: the anticoagulation and risk factors in atrial fibrillation (ATRIA) study. Circulation.

[61] Vazquez E, Sanchez-Perales C, Garcia-Garcia F, Castellano P, Garcia-Cortes MJ, Liebana A, Lozano C (2009). Atrial fibrillation in incident dialysis patients. Kidney Int.

[62] Wizemann V, Tong L, Satayathum S, Disney A, Akiba T, Fissell RB, Kerr PG, Young EW, Robinson BM (2010). Atrial fibrillation in hemodialysis patients: clinical features and associations with anticoagulant therapy. Kidney Int.

[63] Connolly SJ, Ezekowitz MD, Yusuf S, Eikelboom J, Oldgren J, Parekh A, Pogue J, Reilly PA, Themeles E, Varrone J, Wang S, Alings M, Xavier D, Zhu J, Diaz R, Lewis BS, Darius H, Diener HC, Joyner CD, Wallentin L (2009). Dabigatran versus warfarin in patients with atrial fibrillation. N Engl J Med.

[64] Hart RG, Pearce LA, Aguilar MI (2007). Meta-analysis: antithrombotic therapy to prevent stroke in patients who have nonvalvular atrial fibrillation. Ann Intern Med.

[65] Gage BF, Waterman AD, Shannon W, Boechler M, Rich MW, Radford MJ (2001). Validation of clinical classification schemes for predicting stroke: results from the National Registry of Atrial Fibrillation. JAMA.

[66] Goldstein LB, Bushnell CD, Adams RJ, Appel LJ, Braun LT, Chaturvedi S, Creager MA, Culebras A, Eckel RH, Hart RG, Hinchey JA, Howard VJ, Jauch EC, Levine SR, Meschia JF, Moore WS, Nixon JV, Pearson TA, AH (2011). Guidelines for the primary prevention of stroke: a guideline for healthcare professionals from the American Heart Association/American Stroke Association. Stroke.

[67] Ruff CT, Giugliano RP, Braunwald E, Hoffman EB, Deenadayalu N, Ezekowitz MD, Camm AJ, Weitz JI, Lewis BS, Parkhomenko A, Yamashita T, Antman EM (2014). Comparison of the efficacy and safety of new oral anticoagulants with warfarin in patients with atrial fibrillation: a meta-analysis of randomised trials. Lancet.

[68] Hart RG, Eikelboom JW, Brimble KS, McMurtry MS, Ingram AJ (2013). Stroke prevention in atrial fibrillation patients with chronic kidney disease. Can J Cardiol.

[69] Olesen JB, Lip GY, Kamper AL, Hommel K, Køber L, Lane DA, Lindhardsen J, Gislason GH, Torp-Pedersen C (2012). Stroke and bleeding in atrial fibrillation with chronic kidney disease. N Engl J Med.

[70] Herzog CA, Asinger RW, Berger AK, Charytan DM, Díez J, Hart RG, Eckardt KU, Kasiske BL, McCullough PA, Passman RS, Deloach SS, Pun PH, Ritz E (2011). Cardiovascular disease in chronic kidney disease. A clinical update from Kidney Disease: Improving Global Outcomes (KDIGO). Kidney Int.

[71] Elliott MJ, Zimmerman D, Holden RM (2007). Warfarin anticoagulation in hemodialysis patients: a systematic review of bleeding rates. Am J Kidney Dis.

[72] Chan KE, Lazarus JM, Thadhani R, Hakim RM (2009). Warfarin use associates with increased risk for stroke in hemodialysis patients with atrial fibrillation. J Am Soc Nephrol.

[73] Winkelmayer WC, Liu J, Setoguchi S, Choudhry NK (2011). Effectiveness and safety of warfarin initiation in older hemodialysis patients with incident atrial fibrillation. Clin J Am Soc Nephrol.

[74] Marinigh R, Lane DA, Lip GY (2011). Severe renal impairment and stroke prevention in atrial fibrillation: implications for thromboprophylaxis and bleeding risk. J Am Coll Cardiol.

[75] MacMahon S, Collins R (2001). Reliable assessment of the effects of treatment on mortality and major morbidity, II: observational studies. Lancet.

[76] Shah M, Avgil Tsadok M, Jackevicius CA, Essebag V, Eisenberg MJ, Rahme E, Humphries KH, Tu JV, Behlouli H, Guo H, Pilote L (2014). Warfarin use and the risk for stroke and bleeding in patients with atrial fibrillation undergoing dialysis. Circulation.

[77] Carrero JJ, Evans M, Szummer K, Spaak J, Lindhagen L, Edfors R, Stenvinkel P, Jacobson SH, Jernberg T (2014). Warfarin, kidney dysfunction, and outcomes following acute myocardial infarction in patients with atrial fibrillation. JAMA.

[78] Baigent C, Blackwell L, Collins R, Emberson J, Godwin J, Peto R, Buring J, Hennekens C, Kearney P, Meade T, Patrono C, Roncaglioni MC, Zanchetti A (2009). Aspirin in the primary and secondary prevention of vascular disease: collaborative meta-analysis of individual participant data from randomised trials. Lancet.

[79] Antithrombotic Trialists' collaboration (2002). Collaborative meta-analysis of randomised trials of antiplatelet therapy for prevention of death, myocardial infarction, and stroke in high risk patients. BMJ.

[80] Stroke Thrombolysis Trialists' collaboration Impact of treatment delay, age and stroke severity on the effects of intravenous thrombolysis with alteplase in acute ischaemic stroke: individual patient data meta-analysis of randomised trials.

[81] Lee JG, Lee KB, Jang IM, Roh H, Ahn MY, Woo HY, Hwang HW (2013). Low glomerular filtration rate increases hemorrhagic transformation in acute ischemic stroke. Cerebrovasc Dis.

[82] Power A, Fogarty D, Wheeler DC (2013). Acute stroke thrombolysis in end-stage renal disease: a national survey of nephrologist opinion. Nephron Clin Pract.

[83] Palacio S, Gonzales NR, Sangha NS, Birnbaum LA, Hart RG (2011). Thrombolysis for acute stroke in hemodialysis: international survey of expert opinion. Clin J Am Soc Nephrol.

[84] Jauch EC, Saver JL, Adams HP, Bruno A, Connors JJ, Demaerschalk BM, Khatri P, McMullan PW, Qureshi AI, Rosenfield K, Scott PA, Summers DR, Wang DZ, Wintermark M, Yonas H (2013). Guidelines for the early management of patients with acute ischemic stroke: a guideline for healthcare professionals from the American Heart Association/American Stroke Association. Stroke.

[85] Bugnicourt JM, Chillon JM, Massy ZA, Canaple S, Lamy C, Deramond H, Godefroy O (2009). High prevalence of intracranial artery calcification in stroke patients with CKD: a retrospective study. Clin J Am Soc Nephrol.

[86] Jono S, McKee MD, Murry CE, Shioi A, Nishizawa Y, Mori K, Morii H, Giachelli CM (2000). Phosphate regulation of vascular smooth muscle cell calcification. Circ Res.

[87] Block GA, Klassen PS, Lazarus JM, Ofsthun N, Lowrie EG, Chertow GM (2004). Mineral metabolism, mortality, and morbidity in maintenance hemodialysis. J Am Soc Nephrol.

[88] Gutiérrez OM, Mannstadt M, Isakova T, Rauh-Hain JA, Tamez H, Shah A, Smith K, Lee H, Thadhani R, Jüppner H, Wolf M (2008). Fibroblast growth factor 23 and mortality among patients undergoing hemodialysis. N Engl J Med.

[89] Palmer SC, Hayen A, Macaskill P, Pellegrini F, Craig JC, Elder GJ, Strippoli GF (2011). Serum levels of phosphorus, parathyroid hormone, and calcium and risks of death and cardiovascular disease in individuals with chronic kidney disease: a systematic review and meta-analysis. JAMA.

[90] Navaneethan SD, Palmer SC, Craig JC, Elder GJ, Strippoli GF (2009). Benefits and harms of phosphate binders in CKD: a systematic review of randomized controlled trials. Am J Kidney Dis.

[91] Chertow GM, Block GA, Correa-Rotter R, Drüeke TB, Floege J, Goodman WG, Herzog CA, Kubo Y, London GM, Mahaffey KW, Mix TC, Moe SM, Trotman ML, Wheeler DC, Parfrey PS (2012). Effect of cinacalcet on cardiovascular disease in patients undergoing dialysis. N Engl J Med.

[92] Murray AM, Seliger S, Lakshminarayan K, Herzog CA, Solid CA (2013). Incidence of stroke before and after dialysis initiation in older patients. J Am Soc Nephrol.

[93] Toyoda K, Fujii K, Fujimi S, Kumai Y, Tsuchimochi H, Ibayashi S, Iida M (2005). Stroke in patients on maintenance hemodialysis: a 22-year single-center study. Am J Kidney Dis.

[94] Foley RN, Gilbertson DT, Murray T, Collins AJ (2011). Long interdialytic interval and mortality among patients receiving hemodialysis. N Engl J Med.

[95] Culleton BF, Walsh M, Klarenbach SW, Mortis G, Scott-Douglas N, Quinn RR, Tonelli M, Donnelly S, Friedrich MG, Kumar A, Mahallati H, Hemmelgarn BR, Manns BJ (2007). Effect of frequent nocturnal hemodialysis vs conventional hemodialysis on left ventricular mass and quality of life: a randomized controlled trial. JAMA.

[96] Chertow GM, Levin NW, Beck GJ, Depner TA, Eggers PW, Gassman JJ, Gorodetskaya I, Greene T, James S, Larive B, Lindsay RM, Mehta RL, Miller B, Ornt DB, Rajagopalan S, Rastogi A, Rocco MV, Schiller B, Sergeyeva O, Schulman G, Ting GO, Unruh ML, Star RA, Kliger AS (2010). In-center hemodialysis six times per week versus three times per week. N Engl J Med.

[97] Maduell F, Moreso F, Pons M, Ramos R, Mora-Macià J, Carreras J, Soler J, Torres F, Campistol JM, Martinez-Castelao A (2013). High-efficiency postdilution online hemodiafiltration reduces all-cause mortality in hemodialysis patients. J Am Soc Nephrol.

[98] Ishida K, Brown MG, Weiner M, Kobrin S, Kasner SE, Messé SR (2014). Endocarditis is a common stroke mechanism in hemodialysis patients. Stroke.

[99] Santos JP, Hamadeh Z, Ansari N (2012). Cerebrovascular accident secondary to paradoxical embolism following arteriovenous graft thrombectomy. Case Rep Nephrol.

[100] Toyoda K, Fujii K, Ando T, Kumai Y, Ibayashi S, Iida M (2004). Incidence, etiology, and outcome of stroke in patients on continuous ambulatory peritoneal dialysis. Cerebrovasc Dis.

[101] Abramson JL, Jurkovitz CT, Vaccarino V, Weintraub WS, McClellan W (2003). Chronic kidney disease, anemia, and incident stroke in a middle-aged, community-based population: the ARIC Study. Kidney Int.

[102] Weiner DE, Tighiouart H, Vlagopoulos PT, Griffith JL, Salem DN, Levey AS, Sarnak MJ (2005). Effects of anemia and left ventricular hypertrophy on cardiovascular disease in patients with chronic kidney disease. J Am Soc Nephrol.

[103] Tripepi G, Mattace-Raso F, Rapisarda F, Stancanelli B, Malatino L, Witteman J, Zoccali C, Mallamaci F (2010). Traditional and nontraditional risk factors as predictors of cerebrovascular events in patients with end stage renal disease. J Hypertens.

[104] Drüeke TB, Locatelli F, Clyne N, Eckardt KU, Macdougall IC, Tsakiris D, Burger HU, Scherhag A (2006). Normalization of hemoglobin level in patients with chronic kidney disease and anemia. N Engl J Med.

[105] Skali H, Parving HH, Parfrey PS, Burdmann EA, Lewis EF, Ivanovich P, Keithi-Reddy SR, McGill JB, McMurray JJ, Singh AK, Solomon SD, Uno H, Pfeffer MA (2011). Stroke in patients with type 2 diabetes mellitus, chronic kidney disease, and anemia treated with Darbepoetin Alfa: the trial to reduce cardiovascular events with Aranesp therapy (TREAT) experience. Circulation.

[106] Coyne DW (2012). The health-related quality of life was not improved by targeting higher hemoglobin in the Normal Hematocrit Trial. Kidney Int.

[107] Besarab A, Bolton WK, Browne JK, Egrie JC, Nissenson AR, Okamoto DM, Schwab SJ, Goodkin DA (1998). The effects of normal as compared with low hematocrit values in patients with cardiac disease who are receiving hemodialysis and epoetin. N Engl J Med.

[108] Kawamura M, Fijimoto S, Hisanaga S, Yamamoto Y, Eto T (1998). Incidence, outcome, and risk factors of cerebrovascular events in patients undergoing maintenance hemodialysis. Am J Kidney Dis.

[109] Kuo CC, Lee CT, Ho SC, Kuo HW, Wu TN, Yang CY (2012). Haemodialysis and the risk of stroke: a population-based cohort study in Taiwan, a country of high incidence of end-stage renal disease. Nephrology (Carlton).

[110] Iseki K, Fukiyama K (2000). Clinical demographics and long-term prognosis after stroke in patients on chronic haemodialysis. Nephrol Dial Transplant.

[111] Wanner C, Krane V, März W, Olschewski M, Mann JF, Ruf G, Ritz E (2005). Atorvastatin in patients with type 2 diabetes mellitus undergoing hemodialysis. N Engl J Med.

[112] Krane V, Heinrich F, Meesmann M, Olschewski M, Lilienthal J, Angermann C, Störk S, Bauersachs J, Wanner C, Frantz S (2009). Electrocardiography and outcome in patients with diabetes mellitus on maintenance hemodialysis. Clin J Am Soc Nephrol.

[113] Fellström BC, Jardine AG, Schmieder RE, Holdaas H, Bannister K, Beutler J, Chae DW, Chevaile A, Cobbe SM, Grönhagen-Riska C, De Lima JJ, Lins R, Mayer G, McMahon AW, Parving HH, Remuzzi G, Samuelsson O, Sonkodi S, Sci D, Süleymanlar G, Tsakiris D, Tesar V, Todorov V, Wiecek A, Wüthrich RP, Gottlow M, Johnsson E, Zannad F (2009). Rosuvastatin and cardiovascular events in patients undergoing hemodialysis. N Engl J Med.

[114] Holdaas H, Holme I, Schmieder RE, Jardine AG, Zannad F, Norby GE, Fellström BC (2011). Rosuvastatin in Diabetic Hemodialysis Patients. J Am Soc Nephrol.

